# Practice of defensive medicine among surgeons in Ethiopia: cross-sectional study

**DOI:** 10.1186/s12910-023-00979-w

**Published:** 2023-11-08

**Authors:** Eskinder Amare Assefa, Yonas Ademe Teferi, Birhanu Nega Alemu, Abraham Genetu

**Affiliations:** 1 Department of Surgery, Debre Berhan University Asrat Woldeyes Health Science Campus, Debre Berhan, Ethiopia; 2 Department of Surgery, Unit of Cardiothoracic Surgery, Addis Ababa University School of Medicine, Addis Ababa, Ethiopia

**Keywords:** Defensive medicine, Ethiopia, Liability, Litigation, Malpractice, Surgeons

## Abstract

**Background:**

Defensive medicine is physicians’ deviation from standard medical care which is primarily intended either to reduce or avoid medico legal litigation. Although the Federal Ethics Committee review in Ethiopia has shown that applications for medical/surgical error investigation claims are increasing at an alarming rate, there is no study to date done to estimate the degree of defensive practice done by the physicians with an intention of avoiding this increasing legal claim. This study assessed the practice of defensive medicine among highly litigious disciplines (surgery) and described factors associated with its practice.

**Methods:**

Cross sectional quantitative study using online survey questionnaires was conducted to assess the degree of defensive practice and six factors (age, years of experience, specialty, monthly income, place of practice and previous medico legal history) associated with its practice were assessed among surgeons working in Ethiopia.

**Results:**

A total of 430 surgeons directly received an online survey questionnaire and 236 of them successfully completed the questionnaire making the response rate 51.2%. Nearly half of the study participants (51.7%) were aware of the concept of defensive medicine and 174 (74%) reported performing one form of defensive practice. Twenty-nine (12.3%) of the participants have legal dispute history, though only 1.3% of them ended up in penalty. Avoiding high risk procedures was the commonest defensive act performed by 60% of the participants, followed by ordering tests unnecessarily (52.1%). Multinomial logistic regression model showed that there was no association between age of the participant, place of practice, year of experience and defensive practice. This model also showed that cardiothoracic and vascular surgeons perform less defensively than surgeons with other specialty with *P value* of 0.02.

**Conclusion:**

The practice of defensive medicine is widespread among surveyed Ethiopian surgeons and further studies are required to objectively estimate the effect of defensive practice on the health care system of the country. Policy makers need to develop strategy towards decreasing this high rate of defensive practice.

**Supplementary Information:**

The online version contains supplementary material available at 10.1186/s12910-023-00979-w.

## Background

Defensive medicine refers to any deviation from sound medical care given by a physician to avoid or reduce medico-legal liability [1–3]. It could be either positive defensive medicine (termed assurance behavior) or negative defensive medicine (avoidance behavior). Positive defensive medicine involves extra tests or procedures performed with the primary intention of reducing malpractice liability. On the other hand, negative defensive medicine refers to avoiding high-risk patients or procedures [4, 5]. A recent systematic review from Europe, an area where physicians are not financially liable for medical malpractice, has broadened the definition of defensive medicine and included self-protective motives like fear of patient dissatisfaction, fear of overlooking severe illness, and fear of negative publicity as defensive act in addition to fear of legal suit [6].

Defensive medicine (DM) came into practice in the 1970s as the number of malpractice suits started rising geometrically, more frivolous lawsuits were being filed and malpractice insurance premiums began rising. This was with anticipation that it would avoid or markedly reduce malpractice lawsuits [7]. The practice of defensive medicine is common among physicians and its prevalence ranges from 6.7 to 99.8% in various countries [8]. It is practiced by 98% of Japanese gastroenterologists, 93% of high-risk physicians in the United States, and it ranges from 14.9 to 94.5% among Iranian surgeons [4, 9, 10]. The emergence of defensive medicine in Africa is also described by different authors though the concept is not well understood by most physicians in the continent [11–15]. A survey conducted among Sudanese doctors working in obstetrics and gynecology reported that around 71.8% of respondents practice defensively to avoid malpractice claims [13].

Several literatures show there is a steep rise in malpractice complaints and litigation in different parts of Africa [16, 17]. In Ethiopia a study revealed a significant number of surgeons and senior surgical trainees (27.9%) perform at least one serious medical error and nearly 5% of the participants were sued at least once during their practice [18]. Although recent data is lacking analysis done by Wamisho et al. revealed there are an alarmingly increasing number of applications filed for investigation of possible surgical/medical errors in Ethiopia. Thus the Federal Ethics Committee for Health Professionals has made final decisions for a total of 125 medical error claims over a period of seven years from 2011 up to 2017 [19]. This alarmingly increasing number of medical error claims and prevalent malpractice among surgery-related disciplines inevitably brings the practice of DM into the region.

Generally, there are various effects of DM and its practice on the health care system. It can supplement care (performing an additional test or treatment), replace care (referring the patient to another center or physician), or decrease care by avoiding high-risk patients [4]. Defensive medicine can also increase the cost of health care [2, 12, 20]. According to most malpractice scholars, defensive medicine is highly prevalent but it is very difficult to estimate its cost [21]. It is estimated that defensive medicine contributes to as much as $60.2 billion in annual healthcare expenditure in the United States, therefore decreasing defensive medicine will reduce the healthcare cost [11, 21]. Defensive medicine also negatively affects the patient-physician relationship [4, 10].

The main challenge in studying DM is that not every practice to avoid liability results in poor quality of medical care [2]. It is also important to note that a physician can have more than one reason to order a test and it makes it difficult to draw a line between a desire to avoid a lawsuit and a desire to make appropriate treatment for a patient [22]. According to Studdert et al., some of the practices of DM were found to be beneficial [4].

In Ethiopia, there appears to be a paucity of data on defensive medicine and medical malpractice issues. A Federal Ethics Committee showed that 80% of malpractice claims are related to some form of surgery or operation room activities however there is no study conducted in the country to depict the defensive practice of physicians intended to avoid the increasing legal claims. This study was conducted to assess the practice of defensive medicine among the highly litigious discipline in Ethiopia, surgery. The survey also tried to describe the factors associated with the defensive acts of the physicians. The study is unique in the sense that no reports have been previously published from the country with similar objectives. Assessing the baseline level of awareness and practice of surgeons about defensive medicine will give us the necessary data to pass recommendations and design actions to mitigate the negative impact of defensive practice.

## Methods

### Study area and period

The study was conducted among surgeons working in all government and private hospitals in Ethiopia over a period of 2 months from July 1, 2021, G.C up to August 31, 2021, and G.C. All practicing surgeons in different subspecialties were conveniently included in the study because the likelihood of variation in the type of defensive practices performed by the surgeons in different parts of the country.

### Study design, data collection, and sampling

An observational type of cross-sectional study design was used to assess the practice of defensive medicine among physicians practicing surgery. Data was collected from eligible surgeons by using pre-tested structured online survey questionnaires. The survey questionnaire was drafted by the authors after reviewing available literature on the topic around the world (see supplementary file 1). The questionnaire was evaluated by experts and surgeons in different specialties. Two ambiguous questions were modified and one question which deemed unnecessary was removed after the feedback. A pilot study was performed using the survey tool with 10% of the sample size, validity ascertained and necessary modifications made. The online survey questionnaire was sent to each participant through social media networks (telegram and WhatsApp) directly and the responses were collected anonymously using Google form. The questionnaire had 32 in-depth questions divided into four important parts; socio-demographic data, a section assessing awareness towards defensive medicine, specific defensive practice, and factors that influence defensive behavior. Surgeons who refused consent to participate in the survey and those who were not available or accessible during the study period were excluded from the study. Ethical clearance was obtained from AAU College of Health Science and School of Medicine Department of Surgery Research and Ethics Committee. Written informed consent was included on the online survey questionnaire and a cover letter provided basic information about the study.

The estimated minimum required sample size was calculated using a single population proportion formula with a 95% confidence interval (CI) and a 5% margin of error. During the calculation of sample size, we hypothesized the rate of defensive medicine to be comparable with Sudanese doctors, since there was no study done on this topic in the country, and used a population proportion value of 0.718. The study population included about 874 registered surgeons in different specialties and subspecialties, data obtained from the Federal Ministry of Health National Workforce 2020 report. Based on this the sample size was calculated to be 229, but since this was an online survey, we considered the non-response rate to be high and estimated it to be 50%. The final sample size was 460. From a total of 460 participants who were directly accessed through the online survey, 236 of them completely responded to the questionnaire making the response rate 51%.

In this study physicians performing any of the stated assurance or avoidance behaviors were considered as practicing defensive medicine. Assessed assurance behaviors were prescribing more medications than medically indicated, ordering more tests than necessary, writing overzealously/excessively in the patient’s chart, admitting a patient unnecessarily, and suggesting invasive procedures (like a biopsy). Referring a patient unnecessarily, avoiding certain high-risk procedures, and refusing to treat high-risk patients were the avoidance behaviors studied in the survey.

### Data analysis

Data was analyzed using the Statistical Package for Social Sciences (SPSS) version 25.0 software package. Six important predictors of defensive medicine were analyzed using Chi-square (fisher’s exact whenever necessary) probability tests. The six independent variables were; age, years of experience, place of practice, specialty, monthly income, and previous legal dispute history. Associated predictors were further analyzed using binomial and multinomial logistic regression models. During the analysis, a P-value of < 0.05 with a 95% confidence interval was used to judge the significance of the association. Finally, results were displayed using text, frequency tables, and charts.

## Result

### Socio-demographic data

A total of 460 surgeons directly received the online survey questionnaire and 236 of them completely responded to the questionnaire during the survey administration period making the response rate 51%. The majority of the participants were male 205 (86.9%) and the age of the participants ranged from 29 years to 66 years. General surgeons are the most represented specialty in the survey 109 (46.2%) and none of the physicians involved in the survey mentioned having health insurance coverage. Table 1 shows details of the socio-demographic characteristics of participants involved in the survey.

**Table 1:** Socio-demographic characteristics of participants

Socio-demographic characteristics	Number (n)	Percentage (%)
Gender		
Male	205	86.9
Female	31	13.1
Age of participants		
< 30 years	7	3.0
30–35 years	165	69.9
36–40 years	29	12.3
41–45 years	11	4.7
46–50 years	7	3.0
> 50 years	17	7.2
Place of practice		
Addis Ababa	123	52.1
Regional capital city	54	22.9
Other cities or rural parts of Ethiopia	59	25.0
Institution		
Government hospital	138	58.5
Both government and private hospital	92	39.0
Private hospital	6	2.5
Specialty		
General surgery	109	46.2
Orthopedics	34	14.4
Gynecology and obstetrics	29	12.3
Urology	14	5.9
Cardiothoracic/Vascular surgery	13	5.5
Other specialty/subspecialty*	37	
Years of experience	Number (n)	Percentage (%)
< 5 years	171	72.5
5–9 years	40	16.9
10–14 years	7	3.0
15–19 years	7	3.0
>= 20 years	11	4.7
Monthly income in ETB		
<15,000	50	21.2
15,000–29,999	87	36.9
30,000–49,999	64	27.1
>= 50,000	35	14.8
Total	236	100.0

* Other specialty includes: - Pediatric surgery, Hepatobiliary surgery, Colorectal surgery, Endocrine surgery, Plastic and reconstructive surgery

From a total of 236 surveyed doctors 29 (12.3%) of them had directly experienced legal dispute and the majority won the dispute while 3 (10%) of them lost the dispute.

### Awareness of defensive medicine and its impact

**Fig. 1:**
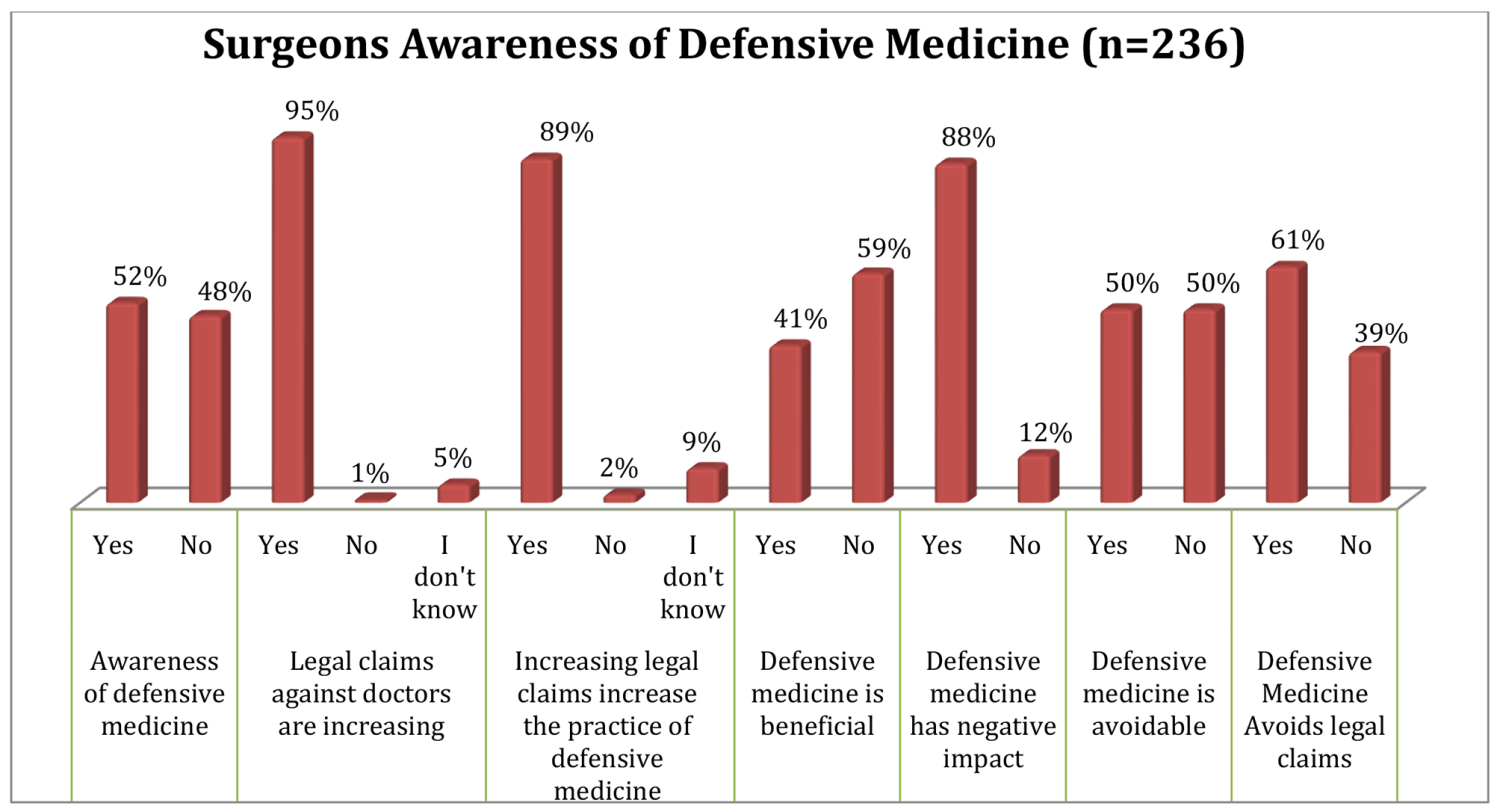
Awareness of surgeons towards defensive medicine

Nearly half (51.7%) of the surgeons who participated in the survey are aware of the concept of DM while the remaining 114 (48.3%) of them reported having never come across the concept. Figure 1 shows the details of the awareness of respondents towards DM and its impact.

### Defensive practice

Nearly three-fourths, 175 (74.2%) of surveyed physicians reported that they have performed one or another form of defensive medicine. Of these respondents, 60 (34.4%) of them admitted to being involved either sometimes or often, while the remaining 114 (65.5%) rarely act defensively. From a total of 236 participants 174 (73.7%) of them detailed their most recent defensive practice and the majority 109 (62.6%) did not remember the time of their most recent defensive act. Table 2 shows the details of recent and specific defensive acts.

**Table 2:** Specific most recent defensive act/s by surgeons

Type of most recent defensive act	Number (n)	Percentage (%)
Ordered CT, MRI, or x-ray	65	37.8
Admitted a patient unnecessarily	14	8.1
Obtained cardiac workup	16	9.3
Ordered another test	17	9.9
Referred a patient to another Physician	23	13.4
Refused to treat a critical patient	8	4.7
Other	29	16.9
**Time of most recent defensive act**		
In the past 1 month	16	9.2
In the past 1 year	49	28.2
Don’t remember the time	109	62.6

#### Negative (avoidance behavior) and positive (assurance behavior) defensive practice

Avoiding high-risk procedures was the commonest of all types of defensive behavior reported by 132 (60.2%) of the surveyed surgeons; being performed either sometimes or rarely. On the other hand, ordering more tests than indicated was the commonest assurance behavior practiced by most of the investigated surgeons (see Table 3).

**Table 3:** Specific most recent defensive act/s by surgeons

Specific defensive practice	Often (%)	Rarely (%)	Never (%)
Ordering more tests than indicated	15 (6.4%)	108 (45.8%)	113 (47.9%)
Prescribed more medications than medically indicated	20 (8.5%)	94 (39.8%)	122 (51.7%)
Written in patient’s chart overzealously/excessively	39 (16.5%)	80 (33.9%)	117 (49.6%)
Referred a patient unnecessarily	7 (3%)	71 (30.1%)	158 (66.9%)
Admitted a patient unnecessarily	2 (0.8%)	102 (43.2%)	132 (55.9%)
Suggested invasive procedures (like biopsy)	10 (4.2%)	75 (31.8%)	151 (64%)
Avoided certain high-risk procedures	26 (11%)	116 (49.2%)	94 (39.8%)
Avoided caring for high-risk patient	8 (3.4%)	78 (33%)	150 (63.6%)

### Factors associated with defensive practice

Respondents were asked about the factors that commonly lead them to act defensively and the majority of them 229 (97.1%) agreed or strongly agreed that fear of medico-legal litigation was the commonest reason. Additionally, significant proportions of participants stated that the experience of medico-legal litigation 206 (87.3%), fear of negative publicity 203 (86%), fear of disciplinary sanction 197 (84.3%), and fear of compensation 193 (81.8%) were all contributors for defensive practice.

### Correlates of the practice of defensive medicine

Six important predictors of defensive practice were identified and analyzed. Age and years of experience were found to have an association with defensive practice with a *p-value* of < 0.0001 (using Chi-square and Fisher’s exact probability testing). The surgeon’s specialty and place of practice were also found to correlate with defensive acts with a *p-value* of 0.001 and 0.004 respectively. Further analysis was carried out using binomial and multinomial logistic regression models and there was no statistically significant association between age of the participant, place of practice, years of experience, and defensive practice among surgeons. The logistic regression model also showed that cardiothoracic and vascular surgeons perform by 90% (AOR: 0.10; 95% CI: 014, 0.705) less defensively compared to other specialties included in the survey. Table 4 shows an association between defensive practice and its correlates.

**Table 4:** Factors associated with the practice of defensive medicine (n = 236)

Variables	Category	N	COR (95% CI)	AOR (95% CI)	P-value
Age of participants	< 30 years	7	0.053(0.006–0.484)	6.40(0.167–245.35)	0.318
	30–35 years	165	0.022(0.005–0.101)	19.21(0.80-460.35)	0.068
	36–40 years	29	0.051(0.009–0.274)	21.32(0.867–524.23)	0.061
	41–45 years	11	0.233(0.034–1.591)	9.90(0.30-326.41)	0.198
	46–50 years	7	0.800(0.061–10.562)	0.93(0.03–28.77)	0.968
	> 50 years	17	Ref.	Ref.	
Place of practice	Addis Ababa	123	1.835(0.893–3.768)	1.911(0.619–5.899)	0.26
	Regional capital city	54	0.442(0.155–1.262)	1.873(0.733–4.876)	0.19
	Other cities or rural parts of Ethiopia	59	Ref.	Ref.	
Specialty	General surgery	109	0.588(0.258–1.34)	0.725(0.218–2.412)	0.6
	Orthopedic surgery	34	0.359(0.111–1.16)	1.189(0.241–5.854)	0.832
	Gynecology and obstetrics surgery	29	0.543(0.175–1.686)	0.843(0.195–3.651)	0.819
	Urology	14	0.833(0.216–3.21)	1.771(0.192–16.348)	0.614
	Cardiothoracic/Vascular surgery	13	6.944(1.609–29.972)	0.1(0.014–0.705)	0.021
	Other specialty (subspecialty)*	37	Ref.	Ref.	
Years of experience	< 5 years	171	0.017(0.002–0.140)	4.936(0.126–193.92)	0.394
	5–9 years	40	0.054(0.006–0.465)	1.989(0.056–70.586)	0.706
	10–14 years	7	0.600(0.031–11.473)	0.370(0.03–28.77)	0.641
	15–19 years	7	0.600(0.031–11.473)	2.135(0.055–83.52)	0.685
	>= 20 years	11	Ref.	Ref.	

*Other specialty includes: - Pediatric surgery, Hepatobiliary surgery, Colorectal surgery, Endocrine surgery, Plastic and reconstructive surgery.

## Discussion

This is the very first study conducted about the practice of defensive medicine among Ethiopian physicians. The survey showed that only half (51.7%) of the participants were aware of the concept of defensive medicine. This is far less than the awareness of physicians in the United Kingdom (UK) where 98% of them were aware of defensive medical practice, but it was higher than the awareness of Sudanese physicians working in Obstetrics and Gynecology where only 42.7% were found to have awareness [5, 13]. However, it can be seen that the notion of defensive medicine in Ethiopia is at its conception.

The practice of defensive medicine is prevalent worldwide; where the rate is 93% among high-risk physicians in the USA, 84.8% among neurosurgeons in South Africa, and 71.8% among Sudanese doctors working in obstetrics and gynecology [4, 11, 13]. Its prevalence is also high among Iranian surgeons where defensive medicine-related behavior ranges from 14.9 to 94.5% [10]. The defensive act is also significantly practiced among the surveyed surgeons in Ethiopia, where 74% of them reported performing one or another form of defensive practice. This figure is comparable with the rate of practice among Italian surgeons where 75% of them practice defensively but it is relatively lower than the study done among doctors in the UK where the prevalence is about 78% and a survey conducted among Japanese gastroenterologists where almost all (98%) of them reported performing some form of defensive practice [5, 9, 23].

Almost all 223 (94.5%) of the sampled surgeons believe that legal claims against doctors are increasing and 29 (12.3%) of them directly experienced legal claims of which only 3 (1.3%) lost the legal dispute. This number is by far less than the rate of litigation among high-risk specialists in the USA where 88% of them have one or another form of legal dispute and it is also lower than the rate of legal dispute among Chinese obstetric and gynecologic surgeons (61%) [4, 24]. This widespread practice of defensive medicine despite a low rate of legal dispute might be due to the physicians’ self-perceived threats rather than actual or existing litigation. On the other hand, the prevalent defensive practice in the country can be due to fear of negative publicity, fear of compensation, or fear of disciplinary sanction which makes them consider a patient as a riskier subject than someone who needs help. Most importantly the high rate of defensive practice by the surveyed physician might be due to the existing brutality of malpractice claim handling mechanisms in the country. In Ethiopia insurance or institutions are not involved in the compensation process for malpractice claims. Federal Ethics Committee for Health Professionals review shows professionals were either permanently or temporarily suspended from practicing, additional training prescribed, or a different degree of warning was given for malpractice claims [19]. Another report in the country also showed that physicians paid money to resolve damage claims by a patient [18]. This is a different way of addressing medical error claims compared to some other African countries like South Africa where insurance and healthcare institutions are involved in the compensation process [17]. It is also in contrast to some of the European medico-legal systems where physicians are not financially liable for medical malpractice [6].

Our study showed that both avoidance and assurance behaviors are significantly performed by the surgeons who responded to the survey. A study done on junior physicians in Egypt showed avoiding high-risk procedures was the commonest form of defensive practice which is in consistent with our study [15]. In this study 60% of the participants mentioned avoiding high-risk procedures; a figure three times higher than physicians in the UK and significantly higher than Sudanese physicians where only 20.5% of them reported avoiding high-risk procedures [5, 13]. Nearly half of the study participants reported ordering a test more than indicated, which is a higher figure compared to the rate among Japanese gastroenterologists (36%) but relatively lower than the rate observed in the USA (59%) [4, 9].

Many kinds of literature in the world showed age and year of experience were strongly associated with defensive practice [5, 9]. However, our study didn’t show any correlation between age, year of experience, and rate of defensive practice. In this study logistic regression model also showed cardiothoracic and vascular surgeons perform less defensively compared to other specialties. But this might not be a true figure since physicians in this specialty represented less in the survey. On the other hand, income was found not to have an association with defensive acts. This was a finding contrary to the study done by *He et al.*. where the rate of defensive acts was relatively higher among low-income physicians [25]. This difference can be due to the lack of awareness of the surveyed doctors in this study where ordering unnecessary tests with the intention of getting additional fees is considered a continuum of standard medical care rather than a defensive act. Unlike many studies, this survey didn’t show any correlation between defensive practice and previous medical dispute history [4, 24]. This could be because most of the participants who experienced legal disputes won their cases and were confident enough to differentiate clinical decisions with legal consequences from unnecessary defensive acts.

## Conclusion

The practice of DM is widespread among surveyed Ethiopian surgeons. Avoiding high-risk procedures and ordering tests more than indicated were the most common types of defensives practiced by the surveyed physicians. The vast majority of Ethiopian surgeons believe that legal dispute among doctors is increasing, but only a minority got penalized due to malpractice. There was no statistically significant association between ages, years of experience, place of practice, previous legal dispute history, and income of a surgeon with defensive practice. The widespread practice of DM despite the low rate of legal dispute might be due to the physicians’ self-perceived threats or due to the brutal treatment of physicians who lost malpractice claims. Educating surgeons about the possible impact of defensive practice on the health care system and awareness creation about the existing high level of self-believed threat of legal suits rather than actual legal consequences can reduce defensive acts and mitigate the negative impact of defensive medicine. Policymakers and other responsible bodies should design strategies towards decreasing defensive practices by surgeons and their negative impact on the heallthcare system. Further studies are required to objectively estimate the effect of defensive practice on the health care system of the country.

### Electronic supplementary material

Below is the link to the electronic supplementary material.

Supplementary Material 1

## Data Availability

All necessary data are available at the hands of the corresponding author upon reasonable request.

## List of abbreviations

AA: Addis Ababa
AAU: Addis Ababa University
CI: Confidence Interval
DM: Defensive Medicine
ETB: Ethiopian Birr
G.C: Gregorian calendar
SSE: Surgical Society of Ethiopia
SD: Standard Deviation
UK: United Kingdom
USA: United States of America
