# Supplementary material for: Practice of defensive medicine among surgeons in Ethiopia: cross-sectional study
Source: BMC Med Ethics. 2023 Nov 8;24:95. doi: 10.1186/s12910-023-00979-w (PMC10634131; doi:10.1186/s12910-023-00979-w)
Supplement: Supplementary file 1 — Supplementary Material 1 [file 12910_2023_979_MOESM1_ESM.docx]

**Questionnaire on Practice of Defensive Medicine Among Surgeons Ethiopia: Cross-Sectional Study**

This questionnaire has four parts; the first one assesses the demographic status of respondents, the second one assesses the knowledge of surgeons towards defensive medicine, the third assesses the specific type of practice of defensive medicine, and the final part assesses factors influencing defensive behaviors and experience on medico-legal suit among surgeons in Ethiopia.

**Part I – Socio-demographic data**

1. Age__________________
2. Gender

Male

Female

1. What is your specialty?

General surgery

Hepatobiliary/Colorectal/Endocrine surgery

Cardiothoracic surgery

Urology

Pediatrics surgery

Neurosurgery

Plastic and reconstructive surgery

Orthopedics

Gynecology and obstetrics

1. Where do you live and practice surgery currently?

Addis Ababa

Regional Capital City

Other city/Rural part of Ethiopia

1. Where do you practice surgery?

Government hospital

Private Hospital

Both

1. Years of experience in Your specialty: ______________________
2. How much is your estimated income per month (in Ethiopian birr)__________________
3. Do you have an insurance cover for any medical error?

Yes

No

**Part II – Awa**r**eness towards defensive medicine and related**

1. Do you think legal claims against doctors are increasing?

Yes

No

I don’t know

1. Do you think increasing legal claims has negative consequences on the healthcare system?

Yes

No

Maybe

1. Have you come across the concept of defensive medicine before?

Yes

No

1. Do you think increasing legal claims against doctors will increase the practice of defensive medicine?

Yes

No

I don’t know

1. Do you think defensive medicine is beneficial?

Yes

No

1. Do you think defensive medicine has a negative impact on the healthcare system? (if no please proceed to question no.8)

Yes

No

1. If “**YES**” what is the possible negative impact on the health care system?

Increase health care cost

Decrease the quality of patient care

Negatively affect doctor-patient relationship

All the three

Other_______________________________________________________

1. Do you think defensive medicine is avoidable?

Yes

No

1. Do you think defensive medicine avoid/reduce legal suit?

Yes

No

**Part III – Practice of specific type of defensive medicine**

1. Have you ever performed any form of defensive type of practice?

Yes

No

Maybe

1. If **“YES**” how often do you perform defensive acts?

Often

Sometimes

Rarely

1. When do you think you performed some form of defensive medicine most recently?

In the past 1 month

In the past 1 year

Don’t remember the time

1. What is your most recent act of defensive practice?

Ordered CT, MRI, or x-ray

Admitted a patient while the patient could have been treated in the outpatient

Obtained cardiac workup

Ordered other tests

Referred patient to another physician

Refused to treat a critical patient

Other____________________________________________________________

1. Do you order more tests than medically indicated?

Often

Rarely

Never

1. Have you prescribed more medications (eg, antibiotics) than medically indicated?

Often

Rarely

Never

1. Have you written in a patient's chart overzealously (excessively) to avoid future medico-legal problems?

Often

Rarely

Never

1. Do you refer patients to other specialists in unnecessary circumstances?

Often

Rarely

Never

1. Have you admitted a patient while it could have been managed as an out-patient?

Often

Rarely

Never

1. Do you suggest invasive procedures (for example, biopsies) to confirm diagnoses more often than the actual clinical indication?

Often

Rarely

Never

1. Have you avoided certain procedures or interventions?

Often

Rarely

Never

1. Have you avoided caring for high-risk patients?

Often

Rarely

Never

**Part IV – Factors influencing defensive behaviors and experience in a legal suit**

1. Have you experienced legal disputes/litigation?

Yes

No

1. If “YES”, did you win the case/dispute?

Yes

No

The case is in progress

1. What factors do you think make a physician act defensively?
2. Fear of medical-legal litigation.

Strongly Agree  Agree  Disagree  Strongly disagree

1. Previous personal experience in medical-legal litigation

Strongly Agree  Agree  Disagree  Strongly disagree

1. Fear of disciplinary sanctions

Strongly Agree  Agree  Disagree  Strongly disagree

1. Fear of a request for compensation

Strongly Agree  Agree  Disagree  Strongly disagree

1. Fear of negative publicity, and loss of image.

Strongly Agree  Agree  Disagree  Strongly disagree
